# Supplementary material for: Time series learning in a many-body Rydberg system with emergent collective amplification
Source: Nat Commun. 2026 Aug 24;17:10118. doi: 10.1038/s41467-026-77083-1 (PMC13597517; doi:10.1038/s41467-026-77083-1)
Supplement: Supplementary file 1 [file 41467_2026_77083_MOESM1_ESM.pdf]

nature portfolio

Peer Review File

Time series learning in a many-body Rydberg system with emergent collective amplification

Corresponding Author: Dr Zongkai Liu

This file contains all reviewer reports in order by version, followed by all author rebuttals in order by version.

Attachments originally included by the reviewers as part of their assessment can be found at the end of this file.

Version 0:

Reviewer comments:

Reviewer #1

(Remarks to the Author)
This manuscript presents a novel experimental study exploring the thermal Rydberg atom vapor as a physical platform for time series prediction and reservoir computing. By encoding time-series data into the amplitude modulation of the coupling laser's Rabi frequency, the authors demonstrate that the system's predictive performance for chaotic and stochastic signals (e.g., the Lorenz attractor and Beijing temperature data) is notably enhanced when operating near a non-equilibrium phase transition, specifically within the bistable regime.

The work is conceptually appealing. It successfully bridges the fields of Rydberg atoms and physical reservoir computing, demonstrating how a many-body quantum system can be harnessed for information processing. The experimental realization of computation enhanced by critical dynamics in a scalable, room-temperature system is a significant result. The manuscript is well-written, and its central point—that collective effects amplify computational capacity—is well supported by the presented experimental data and mean-field theory model.

I recommend publication after the authors address the following comments.

1. The authors state that the raw transmission data T(t_n) are processed with a Savitzky-Golay filter (window size 10, polynomial order 3) before down-sampling and prediction. Standard implementations of Savitzky-Golay filters use a symmetric window that includes both past and future data points relative to the central point. To ensure the robustness of the time-series prediction task, could the authors clarify whether a causal (one-sided) filter was used in the prediction pipeline? If a symmetric filter was applied, please verify (e.g., via a control test using a strictly causal smoothing method) that its use does not inadvertently leak future information into the training phase, which could artificially enhance prediction performance.

2. The experiment demonstrates effective prediction of the Lorenz system, which typically requires the reservoir to possess a "fading memory" of past inputs. The measured relaxation time of the system ($\tau \approx 0.15$ ms, Fig. 4) is comparable to the input modulation period (0.25 ms). It would be insightful for readers from the reservoir computing community if the authors could add a brief comment on the source of the system's effective memory. For instance, does the down-sampling procedure (by a factor of 20) play a role in matching the effective prediction time-step to the system's internal dynamical timescales? A sentence or two clarifying how sufficient input history is retained for the prediction task would be beneficial.

(Remarks on code availability)

Reviewer #2

(Remarks to the Author)
The paper demonstrates reservoir computing in a thermal Rydberg EIT vapor. The input time series is encoded by modulating the coupling field, the probe transmission provides the reservoir output, and a linear regression readout is trained

for time-series prediction. The authors report best performance in a detuning region where the optical response becomes bistable and hysteretic, and they attribute this to an emergent many-body effect near a non-equilibrium phase transition.

While the experimental results are interesting, I find the following shortfalls in the manuscript:

1. Mean-field theory cannot justify “emergent quantum” claims
The theoretical explanation is a mean-field rate-equation model with classical noise after eliminating coherences and factorizing correlations. This framework cannot support claims that quantum many-body resources (correlations/entanglement) drive the computational improvement. As written, the theory supports only a classical statement: a nonlinear bistable dynamical system can improve prediction. The paper should either tone down the “quantum emergent” framing or provide evidence beyond mean-field that quantum many-body dynamics are essential.

2. Generality is not established
The phase-transition-improves-computation thesis is tested on too few tasks and a single encoding/readout pipeline. The current result is a correlation for a specific setup, not a general principle. At minimum, the authors should add standard reservoir benchmarks and capacity metrics (memory capacity, nonlinear capacity, information-processing capacity) and show robustness across window length, modulation depth/bandwidth, and sampling choices.

3. Mechanism is unclear: quantum advantage vs classical operating-point optimization
The output is classical transmission and the readout is linear. Performance gains near bistability could come from classical effects: larger signal swing, longer relaxation (memory), or hysteresis-driven history dependence matched to the task timescales. The authors should control for SNR/bandwidth and compare against a matched classical bistable/nonlinear baseline. Without this, attributing improvement to “quantum” physics is not warranted.

4. Interaction Hamiltonian is under-justified
An all-to-all interaction Hamiltonian with a single parameter is introduced without showing it is appropriate for a thermal vapor with distance-dependent van der Waals/dipolar interactions and spatial/velocity averaging. The paper should provide order-of-magnitude estimates (density, interparticle spacing, blockade radius) and explain how the effective interaction parameter maps to the experimental regime, or clearly label the model as phenomenological with stated limits.

5. Hysteresis is not a reliable measure of nonlinearity
Hysteresis indicates bistability and memory but does not quantify the nonlinear mixing relevant for reservoir computing. The authors should use a proper nonlinearity/computational-quality metric along the operating trajectory, such as nonlinear memory capacity, information-processing capacity, Volterra/NARX kernel estimates, or harmonic/intermodulation distortion under sinusoidal/two-tone modulation.

Minor concerns

* Filtering and segmentation can induce correlations across sub-series and understate uncertainties; leakage checks and block-wise splits are recommended.
* MSE should be complemented with normalized RMSE (relative to target variance) for interpretability.

The experiments suggest an interesting tuning rule: this atomic reservoir predicts best near a bistable regime. Nevertheless, the manuscript’s stronger claim—that emergent quantum many-body criticality is the computational resource—is not supported by the current modeling and analysis. Therefore, the paper’s main presentation does not satisfy the high standard of this journal.

(Remarks on code availability)

Reviewer #3

(Remarks to the Author)
Comments are attached

(Remarks on code availability)

Version 1:

Reviewer comments:

Reviewer #1

(Remarks to the Author)
The authors have carefully addressed the key concerns raised by the reviewers. In particular, the revisions clarify the causality of the prediction pipeline, explicitly separate memory (readout) from nonlinearity (Rydberg dynamics), and provide new benchmark results (Mackey–Glass, NARMA-n) that quantitatively support the central claims. The theoretical framing

has been appropriately tempered, and the limitations of the mean-field model are now clearly stated. The manuscript is improved, and the authors' responses are thorough and satisfactory. I recommend publication in its present form.

(Remarks on code availability)

Reviewer #2

(Remarks to the Author)
I thank the authors for a substantial revision. Several original concerns are now addressed: the framing is toned down, the model is explicitly labeled as a minimal mean-field description, NRMSE is adopted throughout, the causal-filter control rules out leakage, and the new Mackey–Glass and NARMA-n benchmarks strengthen the empirical case. The NARMA contrast (NRMSE < 1 inside vs. > 7 outside the bistable region) is a clean result.

Four issues remain, and I cannot recommend acceptance until they are addressed.

1. The central mechanistic question is not answered. I asked for a matched classical bistable/nonlinear baseline. The authors substitute "the same vapor at a different detuning," which compares a linear regime to a nonlinear one — it does not distinguish Rydberg-collective nonlinearity from any other bistable nonlinearity. A Schmitt trigger, a Kerr-bistable cavity, or a saturable absorber feeding the same linear readout would plausibly reproduce the NARMA contrast. Until this is ruled out, the claim that collective many-body dynamics are the resource is not supported over the weaker claim that any nonlinear front-end would suffice. The authors should either (a) compare quantitatively to published RC benchmarks using non-Rydberg bistable nonlinearities at matched readout dimension, or (b) acknowledge explicitly in the text that the experiment does not distinguish these hypotheses, and revise the claims accordingly.

2. The abstract was not updated as the rebuttal claims. The response states that "quantum" was removed from the abstract. The revised abstract still reads "noisy many-body quantum systems." This should be corrected.

3. Information-processing capacity was requested and not provided. Task-level NRMSE on NARMA does not replace IPC, which is the standard metric for decomposing linear and nonlinear capacity along the operating trajectory. IPC can be computed from data the authors already have. Alternatively, a two-tone intermodulation-distortion measurement of the medium alone would directly quantify the nonlinearity independent of the readout. At least one should be included.

4. The NARMA sweep is incomplete. Figure 3 reports only two detunings (14 vs. 22 MHz). To support the claim that bistability — not nonlinearity in general — is the resource, NARMA NRMSE should be plotted across the same detuning sweep as Fig. 2(j), so the minimum can be seen to align with the hysteresis interval.

Minor: the request for order-of-magnitude estimates supporting the all-to-all approximation (interparticle spacing, blockade radius at $48D_{5/2}$, mapping of $V/\gamma = 100$ to the experiment) was deflected. A few lines in Methods would close the gap.

(Remarks on code availability)

Reviewer #3

(Remarks to the Author)
Authors have addressed the queries.

(Remarks on code availability)
Authors have addressed the queries.

Version 2:

Reviewer comments:

Reviewer #2

(Remarks to the Author)
All my previous comments are well addressed. Therefore, I recommend publication in the current form.

(Remarks on code availability)

Open Access This Peer Review File is licensed under a Creative Commons Attribution 4.0 International License, which permits use, sharing, adaptation, distribution and reproduction in any medium or format, as long as you give appropriate credit to the original author(s) and the source, provide a link to the Creative Commons license, and indicate if changes were made.
In cases where reviewers are anonymous, credit should be given to 'Anonymous Referee' and the source.
The images or other third party material in this Peer Review File are included in the article's Creative Commons license, unless indicated otherwise in a credit line to the material. If material is not included in the article's Creative Commons license and your intended use is not permitted by statutory regulation or exceeds the permitted use, you will need to obtain permission directly from the copyright holder.
To view a copy of this license, visit https://creativecommons.org/licenses/by/4.0/

Reply - “Time series learning in a many-body Rydberg system with emergent collective nonlinearity”

We thank all reviewers for the time and patience with our manuscript. We believe the changes summarised below—including new experimental data, revised theoretical framing, and additional control experiments—represent exactly such a substantial revision. All changes are highlighted in colour in the revised manuscript.
The main improvements are:

- **New benchmark experiments (Reviewers B, C):** We performed new experiments on the Mackey-Glass chaotic time series and the NARMA-n task ($n = 5, 10, 15$). These standard reservoir computing benchmarks quantify the nonlinear capacity of our system. Inside the bistable regime: NRMSE < 1.0 on all NARMA complexities; outside: NRMSE > 7.0 (NARMA-15).
- **Memory mechanism clarified (Reviewer A):** We now clearly separate the two computational ingredients—memory is supplied by the sliding-window readout ($M = 200$, spanning $\sim 6600\,\tau$), while the Rydberg gas provides the nonlinear transformation.
- **Revised framing (Reviewers B, C):** We removed “quantum” from the abstract and revised the manuscript to emphasise emergent collective nonlinearity. The theoretical model is now explicitly labelled as a minimal theoretical model derived from the Lindblad quantum master equation.
- **Causal-filter control (Reviewer A):** We repeated the Lorenz prediction with a strictly causal FIR filter and without any filter, confirming that the preprocessing does not leak future information.
- **Further improvements (Reviewers B, C):** Adopted NRMSE as the error metric throughout; added panel labels to all figures; expanded the Introduction; substantially revised the Discussion section; corrected the fitting-function notation.

We provide detailed point-by-point responses below.

Response to Reviewer A

Comment A0 — This manuscript presents a novel experimental study exploring the thermal Rydberg atom vapor as a physical platform for time series prediction and reservoir computing. By encoding time-series data into the amplitude modulation of the coupling laser's Rabi frequency, the authors demonstrate that the system's predictive performance for chaotic and stochastic signals (e.g., the Lorenz attractor and Beijing temperature data) is notably enhanced when operating near a non-equilibrium phase transition, specifically within the bistable regime.

The work is conceptually appealing. It successfully bridges the fields of Rydberg atoms and physical reservoir computing, demonstrating how a many-body quantum system can be harnessed for information processing. The experimental realization of computation enhanced by critical dynamics in a scalable, room-temperature system is a significant result. The manuscript is well-written, and its central point—that collective effects amplify computational capacity—is well supported by the presented experimental data and mean-field theory model.

I recommend publication after the authors address the following comments.

Reply: We thank the reviewer for the positive assessment and the recommendation for publication. The reviewer's comments have helped us to significantly improve the clarity and rigour of the manuscript. In particular, we have (i) verified that our preprocessing does not introduce information leakage, supported by a causal-filter control experiment, and (ii) clarified the origin of the effective memory in our prediction pipeline. We address both comments in detail below.

Comment A1 — The authors state that the raw transmission data $T(t_n)$ are processed with a Savitzky-Golay filter (window size 10, polynomial order 3) before down-sampling and prediction. Standard implementations of Savitzky-Golay filters use a symmetric window that includes both past and future data points relative to the central point. To ensure the robustness of the time-series prediction task, could the authors clarify whether a causal (one-sided) filter was used in the prediction pipeline? If a symmetric filter was applied, please verify (e.g., via a control test using a strictly causal smoothing method) that its use does not inadvertently leak future information into the training phase, which could artificially enhance prediction performance.

Reply: We thank the reviewer for this important comment. Our prediction pipeline is designed to be strictly causal and free of information leakage. We address the two aspects preprocessing and prediction separately.

Preprocessing. The symmetric Savitzky-Golay filter (window size 10, polynomial order 3) is applied once to the full raw transmission signal for noise reduction. Its window spans only 10 consecutive samples ($\approx 2.5\,\mathrm{ms}$ at our $4\,\mathrm{kHz}$ modulation rate) and acts locally, so it cannot

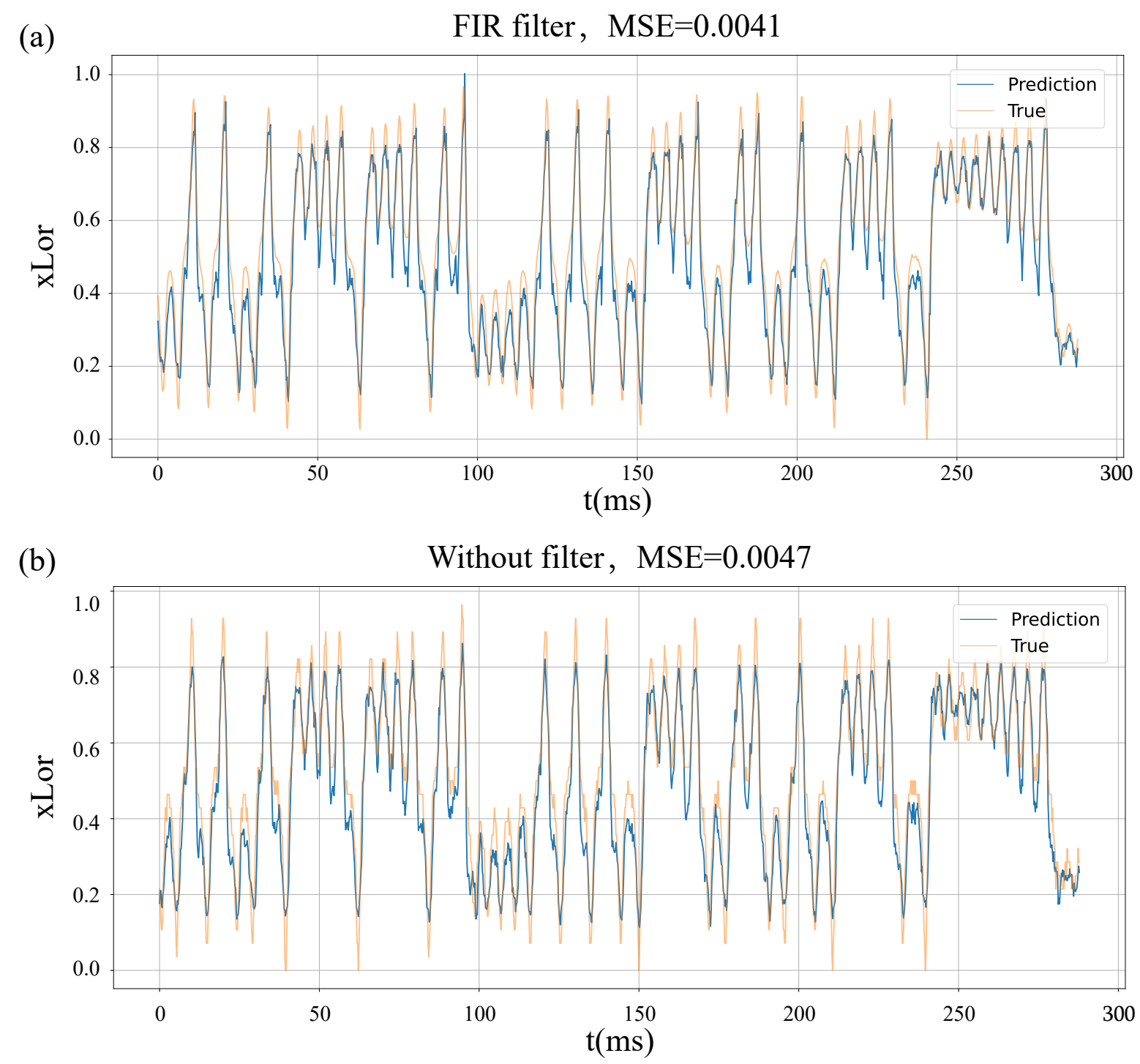

Figure 1: **Causal-filter control experiment for the Lorenz prediction task. (a)** Prediction after replacing the symmetric SG filter with a strictly causal FIR filter. Orange: ground truth x_{Lor}; blue: prediction $\hat{y}$. **(b)** Prediction without any preprocessing filter. Both panels use the same detuning and parameters as the bistable-regime result in Fig. 2(e) of the main text. The prediction quality is consistent across all three filtering choices (symmetric SG, causal FIR, no filter), confirming that the preprocessing does not artificially enhance performance.

transport information over distances relevant to the train/test split. Only after filtering are the data divided into training and test sets.

Prediction. The prediction itself uses a strictly causal sliding window: at each time step, only the M most recent transmission values $\{T(s_{n-M}), \ldots, T(s_n)\}$ are used to predict $x(s_{n+1})$. No future data enter the regression at any stage.

Control experiment. To verify that the symmetric filter does not inadvertently enhance performance, we repeated the Lorenz prediction under two additional conditions: (i) replacing the SG filter with a strictly causal FIR filter of the same effective bandwidth, and (ii) removing all preprocessing entirely. As shown in Fig. 1, the prediction quality is consistent across all three choices. The MSE is in fact slightly *lower* with the causal FIR filter, confirming that the symmetric preprocessing does not inflate performance.

Manuscript revisions:

- **Methods section:** Added “Data processing and causality” paragraph to Methods.
- **Fig. 1 caption, panel (d):** Clarified that the SG filter is applied to the full raw signal

before train/test splitting.

Comment A2 — The experiment demonstrates effective prediction of the Lorenz system, which typically requires the reservoir to possess a "fading memory" of past inputs. The measured relaxation time of the system ($\tau \approx 0.15$ ms, Fig. 4) is comparable to the input modulation period (0.25 ms). It would be insightful for readers from the reservoir computing community if the authors could add a brief comment on the source of the system's effective memory. For instance, does the down-sampling procedure (by a factor of 20) play a role in matching the effective prediction time-step to the system's internal dynamical timescales? A sentence or two clarifying how sufficient input history is retained for the prediction task would be beneficial.

Reply: We thank the reviewer for this insightful question.
Down-sampling by a factor of 20 is strictly a post-processing step applied after the signal has propagated through the ensemble; it does not alter the physical dynamics. The 20 interleaved downsampled series (each shifted by one raw time step) are used to obtain statistical error bars.
The source of memory is the sliding window of length $M = 200$ in the linear readout, not the atomic medium. Each downsampled time step corresponds to $20 \times 0.25\,\mathrm{ms} = 5\,\mathrm{ms}$, so consecutive points are separated by many relaxation times and the atoms retain no relevant fading memory of earlier inputs. Matching the prediction time step to the system's dynamical timescale is therefore not the mechanism behind the prediction performance.
The role of the Rydberg gas is to provide a nonlinear transformation of the input signal. Our new NARMA-n results demonstrate this directly: the sliding window supplies the history, while the bistable dynamics supply the nonlinearity. Inside the bistable regime the system achieves NRMSE < 1.0 across all NARMA complexities; outside it, the processing chain is entirely linear and fails (NRMSE > 7.0 for NARMA-15).
Manuscript revision: We added a section to the discussion clarifying the separation of time scales and the origin of memory in our system.

Response to Reviewer B

Comment B0 — The paper demonstrates reservoir computing in a thermal Rydberg EIT vapor. The input time series is encoded by modulating the coupling field, the probe transmission provides the reservoir output, and a linear regression readout is trained for time-series prediction. The authors report best performance in a detuning region where the optical response becomes bistable and hysteretic, and they attribute this to an emergent many-body effect near a non-equilibrium phase transition.

While the experimental results are interesting, I find the following shortfalls in the manuscript:

[...]

The experiments suggest an interesting tuning rule: this atomic reservoir predicts best near a bistable regime. Nevertheless, the manuscript's stronger claim—that emergent quantum many-body criticality is the computational resource—is not supported by the current modeling and analysis. Therefore, the paper's main presentation does not satisfy the high standard of this journal.

Reply: We thank the reviewer for the careful reading and constructive criticism. The reviewer correctly identifies the central observation of our work: the Rydberg atomic reservoir predicts best near the bistable regime. We agree that this is a physically meaningful and experimentally well-supported finding.

We respectfully clarify that we do not claim a "quantum advantage" over classical computation, nor do we attribute the enhanced performance to purely quantum resources such as entanglement. Our central thesis is that **collective emergent phenomena**—driven by interacting Rydberg atoms—provide a powerful nonlinearity that serves as a physical resource for time-series learning. Whether the underlying description is labelled "quantum" or "classical" does not diminish the physical significance of this observation.

To strengthen the manuscript, we have (i) toned down any wording that could be misinterpreted as claiming quantum advantage, (ii) added new benchmark experiments (Mackey-Glass and NARMA-n) that quantify the nonlinear capacity of the reservoir, and (iii) clarified the scope and limitations of our theoretical model. We address each specific concern in detail below.

Comment B1 — *Mean-field theory cannot justify "emergent quantum" claims*

The theoretical explanation is a mean-field rate-equation model with classical noise after eliminating coherences and factorizing correlations. This framework cannot support claims that quantum many-body resources (correlations/entanglement) drive the computational improvement. As written, the theory supports only a classical statement: a nonlinear bistable dynamical system can improve prediction. The paper should either tone down the "quantum emergent" framing or provide evidence beyond mean-field that quantum many-body dynamics are essential.

Reply: We thank the reviewer for raising this important point.

We respectfully clarify that **we have never claimed that purely quantum resources such as entanglement or strong quantum correlations drive the computational improvement**. To prevent any misinterpretation, we have removed the word "quantum" from the abstract and toned down related phrasing throughout the revised manuscript.

Our theoretical description is a mean-field rate-equation model derived from the Lindblad quantum master equation. The nonlinearity in the mean-field equations arises directly from the interatomic Rydberg interactions, and the framework is conceptually analogous to established mean-field theories of collective phase transitions (e.g., lasing, superconductivity), where a mean-field treatment is widely accepted as a valid description of emergent many-body behaviour. We agree with the reviewer that effects beyond mean-field are not essential here, and we do not rely on them.

Our central thesis is that **collective emergent phenomena**—specifically the nonlinear bistable dynamics generated by the interacting Rydberg ensemble—serve as a physical resource for enhanced learning capability. This statement is independent of whether the underlying mechanism is classified as "quantum" or "classical". The revised manuscript now emphasizes the emergent nonlinear dynamics rather than any purely quantum framing.

Manuscript revisions:

- **Abstract:** Removed "quantum" from the description of emergent phenomena.
- **Theoretical model subsection:** Added text explicitly labelling the model as a minimal theoretical framework derived from the Lindblad master equation, without invoking strong quantum correlations or entanglement as the origin of the observed dynamics.

Comment B2 — *Generality is not established*

The phase-transition-improves-computation thesis is tested on too few tasks and a single encoding/readout pipeline. The current result is a correlation for a specific setup, not a general principle. At minimum, the authors should add standard reservoir benchmarks and capacity metrics (memory capacity, nonlinear capacity, information-processing capacity) and show robustness across window length, modulation depth/bandwidth, and sampling choices.

Reply: We agree with the reviewer that standard reservoir computing benchmarks are essential for evaluating computational capacity. To directly address this concern, we have conducted **new experiments** on two firmly established tasks:

1. **Mackey-Glass (MG) chaotic time series:** We tested our system with delays $\tau_{\mathrm{MG}} = 16$ and 17. Operating inside the bistable region, the reservoir successfully tracks the chaotic dynamics, achieving NRMSE values of 0.34 and 0.28, respectively.
2. **NARMA-n task:** The NARMA benchmark is the standard metric for evaluating a reservoir's ability to retain and nonlinearly mix past inputs. We evaluated complexities $n = 5, 10$, and 15. Inside the bistable region, the system maintains NRMSE < 1.0 across all complexities. Outside the bistable region, where the atomic response is approximately linear, the prediction fails completely (NRMSE > 7.0 for NARMA-15).

These new results reinforce our central physical thesis: the collective nonlinear dynamics in the bistable regime are the computational resource driving the prediction performance. The strong contrast between the bistable and non-bistable regimes—observed consistently across Lorenz, Mackey-Glass, and NARMA tasks—establishes this finding across qualitatively different benchmarks.

We note that exhaustive robustness testing across all possible modulation depths, sampling choices, and window lengths goes beyond the scope of this physics-focused study and constitutes an important direction for future dedicated reservoir computing research.

Manuscript revisions:

- **Results section:** Added Mackey-Glass and NARMA-n benchmark results with corresponding figures.

Comment B3 — *Mechanism is unclear: quantum advantage vs classical operating-point optimization*

The output is classical transmission and the readout is linear. Performance gains near bistability could come from classical effects: larger signal swing, longer relaxation (memory), or hysteresis-driven history dependence matched to the task timescales. The authors should control for SNR/bandwidth and compare against a matched classical bistable/nonlinear baseline. Without this, attributing improvement to "quantum" physics is not warranted.

Reply: We respectfully clarify that **we do not claim a "quantum advantage"** over classical computation. We agree that the output is classical transmission and the readout is linear. Our claim is that the *collective many-body dynamics* of the interacting Rydberg vapour provide a nonlinear transformation that enhances prediction—not that a uniquely quantum resource is at work.

The reviewer suggests controlling for classical effects (signal swing, SNR, relaxation time) and comparing against a matched classical baseline. We argue that **our experiment already provides the most rigorous baseline possible**: the exact same physical system operating outside the bistable region. In this configuration, the atomic density, optical path, laser powers, and detection chain are identical—only the detuning Δ_c changes, moving the system from the collectively nonlinear bistable regime into the approximately linear regime. Any differences in prediction performance are therefore attributable to the presence or absence of the nonlinear response.

Our new NARMA-n results make this point clear. The NARMA task explicitly requires nonlinear transformation of past inputs. Inside the bistable region, where the collective nonlinearity is present, the system achieves NRMSE < 1.0 across all tested complexities ($n = 5, 10, 15$). Outside the bistable region, where the response is approximately linear, and the readout layer is itself strictly linear, the entire processing chain is linear—and the prediction fails catastrophically (NRMSE > 7.0 for NARMA-15). This drastic contrast demonstrates that the computational enhancement originates from the nonlinear capacity provided by the bistable dynamics, which is a well-documented collective many-body effect in Rydberg ensembles.

We have revised the manuscript to make these points explicit and to remove any wording that could be misinterpreted as claiming quantum advantage:

Manuscript revision:

- **Abstract**: Removed "quantum" in "Using the Lorenz time series and temperature data as examples, our work demonstrates how emergent phenomena enhance the capability of noisy many-body systems for data processing and forecasting."
- Added new experimental results for Mackey-Glass and NARMA-n task to the results section
- Added clarification to the theoretical model subsection

Comment B4 — *Interaction Hamiltonian is under-justified*
An all-to-all interaction Hamiltonian with a single parameter is introduced without showing it is appropriate for a thermal vapor with distance-dependent van der Waals/dipolar interactions and spatial/velocity averaging. The paper should provide order-of-magnitude estimates (density, interparticle spacing, blockade radius) and explain how the effective interaction parameter maps to the experimental regime, or clearly label the model as phenomenological with stated limits.

Reply: We thank the reviewer for this comment. As the reviewer suggests, we classify our theoretical framework as a **minimal theoretical model** in the revised manuscript.
The all-to-all interaction Hamiltonian with a single effective coupling parameter is a standard mean-field approximation widely used in the literature to capture the essential collective bistable dynamics of driven-dissipative Rydberg ensembles (Refs. [26–36] in the manuscript). Its primary purpose is not to provide a fully microscopic, first-principles description that resolves distance-dependent van der Waals potentials or velocity-class averaging. Rather, it is designed to **minimally reproduce the macroscopic phase transition and bistable regime** that are relevant for the reservoir computing response.
We emphasise that the key physical ingredient captured by the model—the emergence of bistability and hysteresis from collective interatomic interactions—is robust and does not depend on microscopic details of the interaction potential. A more detailed treatment incorporating distance-dependent interactions and thermal averaging would refine quantitative predictions but is not expected to change the qualitative phenomenology. We consider such extensions an interesting direction for future work.
We have updated the "Theoretical model" section to make the scope and limitations of the model fully transparent:
Manuscript revision: We added a clarification to the theoretical model subsection

Comment B5 — *Hysteresis is not a reliable measure of nonlinearity*
Hysteresis indicates bistability and memory but does not quantify the nonlinear mixing relevant for reservoir computing. The authors should use a proper nonlinearity/computational-quality metric along the operating trajectory, such as nonlinear memory capacity, information-processing capacity, Volterra/NARX kernel estimates, or harmonic/intermodulation distortion under sinusoidal/two-tone modulation.

Reply: We agree with the reviewer that hysteresis, while being a strong physical signature of bistability, does not by itself quantify the nonlinear mixing capacity relevant for reservoir computing.

To provide exactly the kind of rigorous computational metric the reviewer requests, we have performed **new experiments using the NARMA-n benchmark**. The NARMA task is the established standard in the reservoir computing community for evaluating nonlinear memory capacity: it requires the system to retain *and* nonlinearly mix past inputs, and cannot be solved by any purely linear processing chain. We evaluated complexities $n = 5, 10$, and 15.
The results explicitly quantify the nonlinear capacity of our reservoir along the operating trajectory:

- **Inside the bistable region**, where the hysteresis loop indicates strong collective nonlinearity, the system achieves NRMSE < 1.0 across all tested NARMA complexities, confirming substantial nonlinear mixing capacity.
- **Outside the bistable region**, where the atomic response is approximately linear, the NRMSE diverges drastically (exceeding 7.0 for NARMA-15), demonstrating the collapse of nonlinear capacity.

These results establish a direct, quantitative link between the physical operating regime (characterised by the hysteresis loop) and the computational nonlinear capacity measured by a standard reservoir benchmark.
Manuscript revisions in the results section: Added NARMA-n benchmark results as a quantitative measure of nonlinear capacity.

Comment B6 — *Minor concerns:*

1. Filtering and segmentation can induce correlations across sub-series and understate uncertainties; leakage checks and block-wise splits are recommended.
2. MSE should be complemented with normalized RMSE (relative to target variance) for interpretability.

Reply:

1. **Filtering and segmentation:** The Savitzky-Golay filter (window size 10, polynomial order 3) is applied once to the full raw signal before any train/test splitting. Its window spans only 10 consecutive samples ($\approx 2.5\,\mathrm{ms}$), which is far too short to transport information across the training/test boundary. The prediction itself uses a strictly causal sliding window. As an additional control, we repeated the prediction with a strictly causal FIR filter and without any filter (see our response to Comment A1 and Fig. 1), obtaining consistent results. We are therefore confident that the preprocessing does not introduce correlations that understate uncertainties.
2. **Normalized RMSE:** We thank the reviewer for this suggestion. We have replaced MSE with the normalised root mean square error (NRMSE), defined as $\mathrm{NRMSE} = \sqrt{\mathrm{MSE}}/\sigma_{\mathrm{target}}$, throughout the revised manuscript. This provides the interpretability the reviewer requests and is consistent with standard reporting in the reservoir computing literature.

Manuscript revisions: Replaced MSE with NRMSE as the primary error metric throughout the manuscript, including in Fig. 2(j) and Fig. 3(d).

Response to Reviewer C

Comment C0 — Authors address a very interesting avenue of Rydberg systems exhibiting emergent phenomena for time series prediction. Lorenz and temperature data are used to demonstrate enhanced learning capability in a many body system. The problem being addressed is of interest to a broad audience. However, the manuscript still lacks clear and sound physical interpretations of the obtained results. Before recommending for publication, below are my queries:

Reply: We thank the reviewer for recognising the broad interest of our work and for the constructive queries. We agree that the original manuscript benefits from clearer physical interpretations. In the revised version we have (i) strengthened the Introduction by emphasising the nonlinear response and emergent dynamics of Rydberg systems, (ii) added new benchmark experiments (Mackey-Glass and NARMA-n) that quantify the nonlinear capacity of the reservoir, (iii) clarified the scope and limitations of our theoretical model, and (iv) substantially revised the Discussion section. We address each query in detail below.

Comment C1 — The introduction is well written but mininal emphasis is given to the nonlinearity and Rydberg systems exhibiting the emergent dynamics.

Reply: We thank the reviewer for this constructive comment. We have revised the Introduction to place stronger emphasis on the nonlinear response and emergent dynamics in Rydberg systems. In particular, we now explicitly highlight that thermal Rydberg vapours host strong, long-range interatomic interactions and competing drive-dissipation processes, which give rise to a rich variety of emergent non-equilibrium phenomena. These collective effects produce a highly nonlinear optical response (manifesting as bistability and hysteresis), which is exactly the physical resource our work exploits for reservoir computing.

This expanded discussion better motivates why Rydberg systems are a particularly promising platform: they provide *tunable* nonlinearity whose strength can be controlled by the laser detuning, enabling a direct comparison between linear and nonlinear operating regimes within the same physical system.

Manuscript revisions: Expanded discussion of Rydberg nonlinearity and emergent dynamics in the introduction.

Comment C2 — Its better to have subnames to figures for more clarity. Otherwise, its unclear at times which figure is referred.

Reply: We thank the reviewer for this suggestion. We have added explicit panel labels (a), (b), (c), . . . to Fig. 1 and updated all corresponding references in the main text to cite specific panels, improving clarity throughout.

Manuscript revisions: Added explicit panel labels to Fig. 1 and updated all figure references in the main text.

Comment C3 — Authors mention fig 2b is outside bistable regime and c and d are in bistable region. But doesn't explain or justify the spectral features.

Reply: We thank the reviewer for this valuable comment. We have added a clear explanation and justification for the features that distinguish the bistable and non-bistable regimes in the revised manuscript. There are three key aspects:

1. **Definition of the bistable region:** The detuning Δ_c of the coupling laser is the control parameter. The bistable region is defined by the hysteresis loop in Fig. 2(i), formed by scanning Δ_c in the two opposite directions. When Δ_c lies within the shaded hysteresis interval, the system operates in the bistable regime [Figs. 2(c) and 2(d)]; when Δ_c lies outside, it operates in the non-bistable, approximately linear regime (Fig. 2(b)).

2. **Signal characteristics:** Inside the bistable region, the collective nonlinear response produces a markedly larger peak-to-peak amplitude and higher signal-to-noise ratio (SNR) in the time-domain transmission signal. This is a direct consequence of the steep, nonlinear dependence of the Rydberg population on the driving field within the hysteresis loop: small changes in the input modulation are amplified into large changes in transmission.

3. **Prediction performance:** The NRMSE of the Lorenz prediction [Fig. 2(j)] is markedly reduced within the bistable regime. This enhancement cannot be attributed solely to an increased signal-to-noise ratio; rather, it arises from the nonlinear transformation induced by the bistable dynamics. This interpretation is independently supported by our new NARMA-n results, where the system, when operated outside the bistable regime (i.e., in a linear response regime), fails entirely to produce accurate predictions despite exhibiting comparable signal levels.

Manuscript revisions:

- **Revised Figure 2 caption:** "The detuning is chosen such that the system is out of (panel (b), Δ_c outside the hysteresis region in panel (i)) and within the bistable region (panels (c)–(d), Δ_c inside the hysteresis region in panel (i))."
- **Added text in the Results section** (near the description of Fig. 2): "Specifically, the bistable region is defined by the closed hysteresis interval formed by the two scan directions of the coupling laser (shaded interval in panel (i)). When Δ_c lies within this shaded interval, the system operates within the bistable region (e.g., $\Delta_c = 12, 14\,\mathrm{MHz}$), whereas detunings outside this interval (e.g., $\Delta_c = 10\,\mathrm{MHz}$) correspond to the non-bistable regime."

Comment C4 — Authors state "no nonlinear activation is used in this readout layer, so all non linear processing of input orignates from the dynamics of the Rydberg vapor". This statement requires a clearer explanation.

Reply: We thank the reviewer for this comment and agree that the statement deserves a clearer explanation. We have revised the manuscript accordingly.

Our readout layer exclusively employs linear operations: least-squares regression and matrix multiplication. No nonlinear activation functions are applied at any stage. Because the readout is strictly linear, it cannot perform any nonlinear mixing on its own. It follows that **all nonlinear processing of the input signal must originate from the physical dynamics of the Rydberg vapour**.

Our new NARMA-n benchmark results provide direct evidence for this conclusion. The NARMA task explicitly requires nonlinear mixing of past inputs and cannot be solved by any purely linear system. Since our readout is strictly linear, the successful prediction of NARMA-15 inside the bistable region (NRMSE < 1.0) constitutes direct proof that the Rydberg vapour provides the necessary nonlinear computational capacity. Conversely, outside the bistable region—where the atomic response is approximately linear—the entire processing chain becomes linear and the prediction fails (NRMSE > 7.0).

Manuscript revision in the results section: "It is noted that no nonlinear activation functions are used in the readout layer. The readout operations are limited to linear regression (least squares method) and matrix multiplication, which are strictly linear calculations. Therefore, all the nonlinear processing of the input signal observed in our experiments originates from the intrinsic dynamics of the Rydberg vapor, which is characterized by the hysteresis loop in the system's response (Fig. 2i)."

Comment C5 — In the Methods section, a two-level model is used to describe the nonlinear dynamics. This is a very crude approximation for a thermal Rydberg EIT system. Ref. 33 clearly establishes that two-level dynamics cannot fully capture the emergent collective phenomena. The limitations of this approximation should be explicitly discussed.

Reply: We thank the reviewer for this careful observation and fully agree that a two-level model is a simplification of the full three-level EIT ladder scheme in a thermal vapour.

The two-level reduction is obtained by adiabatically eliminating the intermediate state, which is justified when the probe Rabi frequency and decoherence rates of the intermediate level are much smaller than its detuning. Under these conditions the three-level dynamics reduce to an effective two-level driven-dissipative system with a collectively enhanced nonlinearity. This is a standard procedure widely used in the Rydberg EIT literature (Refs. [26–36]).

Regarding Ref. 33: we fully agree with its findings that, at very high Rydberg densities, the two-level picture becomes insufficient and additional collective phenomena emerge that require a more detailed treatment. However, in our experimental regime the Rydberg density is comparatively low, which physically justifies the approximation. Importantly, the two-level mean-field model **successfully reproduces** both the experimentally observed bistability and the qualitative dependence of the prediction error on detuning—confirming that it captures the essential physics relevant to our investigation.

As also discussed in our response to Comment B5, our model is intentionally designed as a **minimal theoretical model**. Its goal is not a fully microscopic description, but to identify the macroscopic emergent features (bistability, hysteresis) that serve as computational resources for

the reservoir. We have added an explicit discussion of the model's scope and limitations in the revised "Theoretical model" section in methods.
Manuscript revisions in the theoretical model subsection: Added explicit discussion of the two-level approximation, its justification, and its limitations in methods.

Comment C6 — The authors do not include Doppler averaging or the role of different velocity classes involved in the interaction that leads to an effective many body global coupling. The implications of neglecting velocity-dependent effects on the collective dynamics and learning performance should be discussed.

Reply: We thank the reviewer for raising this point. Doppler averaging and velocity-dependent interactions are indeed present in any thermal vapour experiment and can, in principle, modify the collective dynamics and the effective many-body coupling.
However, there is direct evidence that these effects are not the primary driver of the learning performance in our system: our mean-field model, which does *not* include Doppler averaging or velocity-class-dependent interactions, already reproduces the experimentally observed dependence of the prediction error on detuning—including the sharp improvement inside the bistable region. This agreement indicates that the dominant mechanism is the collective bistability arising from the interatomic Rydberg interactions. The Doppler effect may quantitatively shift the boundaries of the bistable region, but it does not qualitatively alter the nonlinear response that our reservoir exploits.
We have added a remark in the revised "Theoretical model" section noting that Doppler effects are omitted in the present minimal theoretical model, and that incorporating velocity-dependent dynamics and their impact on the effective global coupling represents a natural refinement for future, more microscopic treatments.
Manuscript revisions in theoretical model subsection: Added remark on the omission of Doppler averaging and its expected minor impact on the qualitative phenomenology, however, interesting to investigate in future experiments.

Comment C7 — In the Methods section on experimental analysis of relaxation times, the fitting function is written as $A\exp\{(-t/\tau)+B\}$. It is unclear whether the constant B is inside the exponential. If so, the interpretation of the extracted relaxation times needs clarification.

Reply: We thank the reviewer for this careful comment. The constant B is **outside** the exponential; the correct fitting function is $A\exp(-t/\tau)+B$, where B accounts for a constant offset in the transmission baseline. The relaxation time τ is therefore extracted from the exponential decay alone and its interpretation is unaffected. We have corrected the expression in the Methods section to remove the ambiguity.
Manuscript revision in method section: "This allows us to obtain the relaxation time by fitting the function $A\exp(-t/\tau)+B$ to every period of the transmission signal... "

Comment C8 — Discussion section is relatitvely weak in justifying the results.

Reply: We thank the reviewer for this feedback and agree that the discussion section needed strengthening. We have substantially revised it to provide clearer and more rigorous physical interpretations. The key improvements are:

1. **Separation of memory and nonlinearity:** We now clearly identify the two distinct computational resources and their origins. The memory of past inputs is supplied by the sliding-window readout ($M = 200$ downsampled points, spanning $\sim 1\,\mathrm{s}$), while the nonlinear transformation is provided by the collective bistable dynamics of the Rydberg vapour. This separation is directly demonstrated by our new NARMA-n results: the sliding window provides the required history, but the prediction succeeds only when the vapour operates in the nonlinear bistable regime.

2. **New benchmark results:** The revised discussion now references the new benchmarks, which quantify the system's forecasting and nonlinear capacity, respectively. The stark contrast between bistable (NRMSE < 1.0) and non-bistable (NRMSE > 7.0 for NARMA-15) regimes provides the quantitative evidence that was previously missing.

3. **Future directions:** We outline concrete next steps, including time-multiplexing and feedback schemes to improve prediction performance, as well as systematic investigations of the relationship between relaxation time, bistable dynamics, and learning capacity.

Manuscript revisions in discussion section: Substantially rewritten to clarify the physical mechanism, incorporate new benchmark results, and strengthen the interpretation.

Reply - “Time series learning in a many-body Rydberg system with emergent collective nonlinearity”

We thank all reviewers for the time and patience with our manuscript. We believe that the revised version fully meets the criteria of Nature Communications, and all modifications are highlighted in colour.
We provide detailed point-by-point responses below.

Response to Reviewer A

Comment A0 — The authors have carefully addressed the key concerns raised by the reviewers. In particular, the revisions clarify the causality of the prediction pipeline, explicitly separate memory (readout) from nonlinearity (Rydberg dynamics), and provide new benchmark results (Mackey–Glass, NARMA-n) that quantitatively support the central claims. The theoretical framing has been appropriately tempered, and the limitations of the mean-field model are now clearly stated. The manuscript is improved, and the authors' responses are thorough and satisfactory. I recommend publication in its present form.

Reply: We thank reviewer for this recommendation.

Response to Reviewer B

Comment B0 — I thank the authors for a substantial revision. Several original concerns are now addressed: the framing is toned down, the model is explicitly labeled as a minimal mean-field description, NRMSE is adopted throughout, the causal-filter control rules out leakage, and the new Mackey–Glass and NARMA-n benchmarks strengthen the empirical case. The NARMA contrast (NRMSE < 1 inside vs. > 7 outside the bistable region) is a clean result.
Four issues remain, and I cannot recommend acceptance until they are addressed.

Reply: We thank the reviewer for the positive comments and constructive feedback. We will address the remaining issues in detail below.
We highlight that after reevaluation, the NRMSE values where wrongly normalized before. This is why we obtained values much larger than 1 in the previous version. We have now corrected this error, and the NRMSE values are now properly normalized and fall within the expected range of around 0 to 1. Nevertheless, the qualitative trends and conclusions remain unchanged, and the NARMA contrast is still a clean result.

Comment B1 — The central mechanistic question is not answered. I asked for a matched classical bistable/nonlinear baseline. The authors substitute "the same vapor at a different detuning," which compares a linear regime to a nonlinear one — it does not distinguish Rydberg-collective nonlinearity from any other bistable nonlinearity. A Schmitt trigger, a Kerr-bistable cavity, or a saturable absorber feeding the same linear readout would plausibly reproduce the NARMA contrast. Until this is ruled out, the claim that collective many-body dynamics are the resource is not supported over the weaker claim that any nonlinear front-end would suffice. The authors should either (a) compare quantitatively to published RC benchmarks using non-Rydberg bistable nonlinearities at matched readout dimension, or (b) acknowledge explicitly in the text that the experiment does not distinguish these hypotheses, and revise the claims accordingly.

Reply: We thank the reviewer for this important comment, and we agree with its substance. We have addressed it in two complementary ways: by sharpening the mechanistic analysis in the manuscript, and by moderating the claims accordingly.
First, we agree that contrasting two detunings only distinguishes a linear from a nonlinear operating regime and does not, by itself, single out Rydberg collective many-body nonlinearity from a generic classical bistable nonlinearity. Our revised analysis (new Methods section "Origin of the learning performance enhancement", with the new gain/SNR and IPC figures) now identifies the computational resource explicitly: near the bistable boundary, the collective susceptibility $\mathcal{G} = \partial\langle T\rangle/\partial\Omega_c$ is enhanced by roughly thirty-fold, while the signal-to-noise ratio of the transfer curve stays essentially flat. This gain near the phase transition is what maps the input onto

a near-linear basis that the linear readout can decode. We therefore agree that the underlying computational principle—a large effective gain near a fold bifurcation—is general, and that comparable performance could in principle be obtained with other bistable nonlinear front-ends feeding an identical linear readout.

Importantly, conceding this does not weaken the contribution of the work. We make no claim that the Rydberg platform is uniquely necessary or quantitatively superior to such alternatives, and we have removed any wording that implied this. What the work demonstrates is that (i) an *emergent collective many-body phase transition* can serve as this computational resource, (ii) it is continuously and reversibly tunable through a single experimental control parameter, the coupling detuning, and (iii) a minimal mean-field model, together with a complete mechanistic dissection (gain, SNR, IPC, and memory horizon), connects the collective physics to the measured prediction performance. A quantitative head-to-head benchmark against engineered non-Rydberg bistable reservoirs at matched readout dimension is a worthwhile dedicated study, which we now flag explicitly as a direction for future work.

Following the reviewer's option (b), we have moderated the claims in the manuscript text accordingly.

Manuscript revisions:

- **Conclusion:** Added: "We stress that this gain mechanism is general rather than specific to Rydberg systems [refs]; its distinctive realisation here is as a many-body phase transition that is continuously tunable through a single control parameter."
- **Conclusion (first paragraph):** Added "in Rydberg atomic systems" to the sentence establishing the link between emergent collective phenomena and learning capability.
- **Methods/Discussion:** The gain, SNR, and IPC analyses (see also our replies to the points below) make the operative mechanism explicit and the corresponding claims precise.

Comment B2 — The abstract was not updated as the rebuttal claims. The response states that "quantum" was removed from the abstract. The revised abstract still reads "noisy many-body quantum systems." This should be corrected.

Reply: We thank you for pointing out this oversight, and the word "quantum" has been deleted from the abstract.

Manuscript revisions:

- **Abstract:** The phrase "noisy many-body quantum systems." is revised to "noisy many-body systems.".

Comment B3 — Information-processing capacity was requested and not provided. Task-level NRMSE on NARMA does not replace IPC, which is the standard metric for decomposing linear and nonlinear capacity along the operating trajectory. IPC can be computed from data the authors already have. Alternatively, a two-tone intermodulation-distortion

measurement of the medium alone would directly quantify the nonlinearity independent of the readout. At least one should be included.

Reply: We agree that task-level NRMSE on NARMA does not substitute for the information processing capacity, and we now provide the IPC explicitly (new figure "Information processing capacity of the Rydberg reservoir" and the Methods section "Origin of the learning performance enhancement"). It is computed directly from the existing discrete-step NARMA dataset, resolved by polynomial degree d and time lag k, using Hermite polynomial targets (orthogonal for the Gaussian distributed random input) orthogonalised by QR decomposition to avoid double-counting capacity across channels.
The informative result lies not in the *total* capacity, which is comparable across detunings, but in its *composition*. Inside the bistable region ($\Delta_c = 14\,\mathrm{MHz}$) the capacity is almost entirely linear ($d = 1$) across all lags. Outside ($\Delta_c = 8\,\mathrm{MHz}$) roughly half of the capacity resides in the quadratic channel ($d = 2$): the input is encoded through the nonlinear tail of the EIT hysteresis loop, in a basis that the linear readout cannot invert. The degree-resolved capacity therefore tracks the V-shaped NRMSE and decomposes the linear versus nonlinear capacity along the operating trajectory, exactly as the reviewer requested. We selected IPC over the two-tone intermodulation alternative because it is computable from the data already in hand and additionally resolves the lag structure, which shows that the memory available to the readout is bounded by the sliding window $M = 200$ rather than by the vapour dynamics.
Manuscript revisions:

- **Methods:** Added the section "Origin of the learning performance enhancement" with the degree- and lag-resolved IPC (new IPC figure) and its detuning dependence.
- **Discussion:** Substantially revised the Discussion section to contextualize the IPC results and clarify the role of the readout layer.

Comment B4 — The NARMA sweep is incomplete. Figure 3 reports only two detunings (14 vs. 22 MHz). To support the claim that bistability — not nonlinearity in general — is the resource, NARMA NRMSE should be plotted across the same detuning sweep as Fig. 2(j), so the minimum can be seen to align with the hysteresis interval.

Reply: We agree, and we have added the full sweep. The new figure "NARMA performance and the readout-defined memory horizon" reports the NARMA-n NRMSE as a function of the coupling detuning Δ_c over the same range as Fig. 2(j), for $n = 5, 10, 15, 100, 200, 300, 500$. For every task within the readout memory horizon ($n \leq 200$), the NRMSE reproduces the V-shape of Fig. 2(j), with its minimum located inside the hysteresis interval; the NARMA optimum thus aligns with the bistable window, as the reviewer requested. As an additional control, for $n \geq 300$—which exceeds the sliding window $M = 200$—the error collapses to the trivial baseline (NRMSE ≈ 0.9) at every detuning, confirming that the operative memory horizon is the readout window and not the physical relaxation of the vapour.
Manuscript revisions:

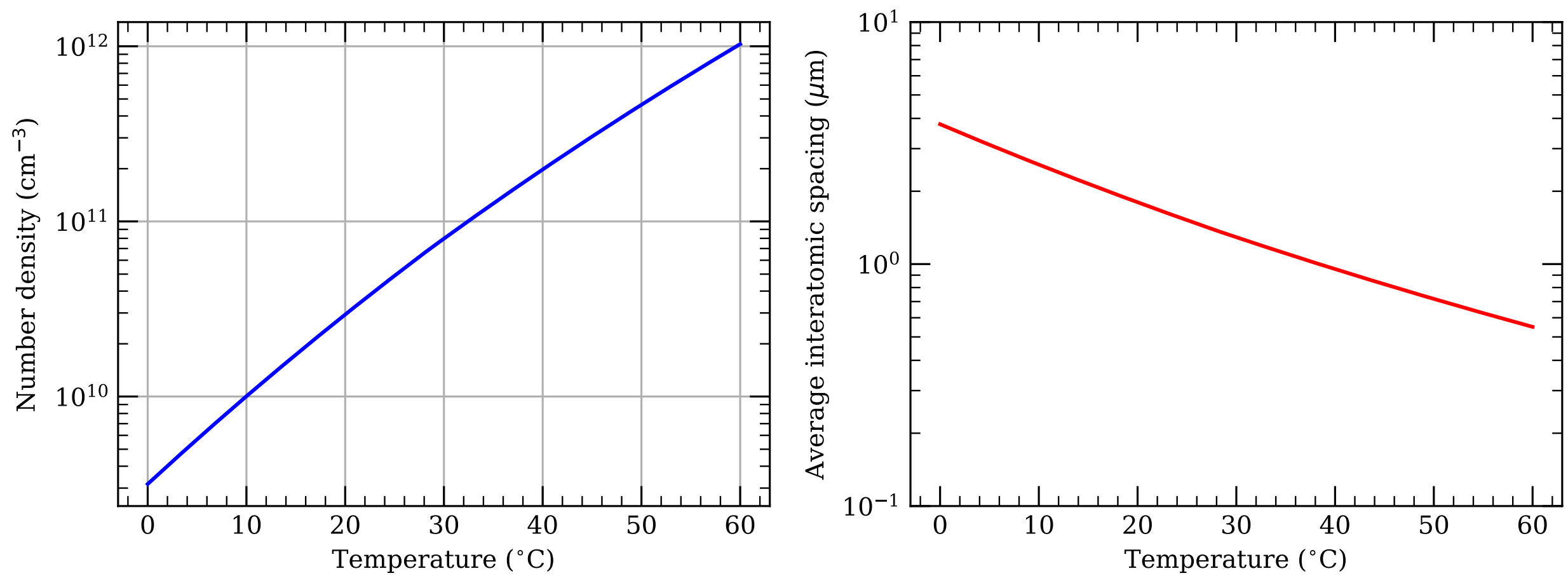

Figure 1: Number density (left panel) and mean interaction distance (right panel) of cesium vapor at different temperatures.

- **Methods:** Added the NARMA-n detuning-sweep figure (NRMSE versus Δ_c for $n = 5, \ldots, 500$) and accompanying discussion.

Comment B5 — Minor: the request for order-of-magnitude estimates supporting the all-to-all approximation (interparticle spacing, blockade radius at 48$D_{5/2}$, mapping of V/γ = 100 to the experiment) was deflected. A few lines in Methods would close the gap.

Reply: We agree, and we have added the requested estimates as a short paragraph in the Methods ("Validity of the all-to-all approximation"). Using the alkali-Rydberg-calculator [1], at the operating temperature $47.0\,°\mathrm{C}$ the caesium density is $n \approx 3.61 \times 10^{11}\,\mathrm{cm}^{-3}$, giving a mean interatomic spacing $a \approx 0.8\,\mu\mathrm{m}$ (Fig. 1). The blockade radius of the $48D_{5/2}$ pair state is $R_\mathrm{b} \approx 2\,\mu\mathrm{m}$ to $3\,\mu\mathrm{m}$ (Fig. 2). Since $R_\mathrm{b} \gtrsim a$, of order ten to several tens of atoms lie within a single blockade volume and respond collectively—precisely the regime in which the all-to-all mean-field treatment is a faithful minimal description. The dimensionless ratio $V/\gamma = 100$ used in the model is consistent with this picture, the van der Waals interaction at the mean spacing ($\sim 10^2$–10^3 MHz) far exceeding the effective linewidth ($\sim$ MHz). The model fixes such dimensionless ratios so as to reproduce the macroscopic bistability rather than to resolve the distance-dependent interaction potential, as we now state explicitly.

Manuscript revisions:

- **Methods:** Added the paragraph "Validity of the all-to-all approximation" with density, interatomic spacing, blockade radius, and the V/γ mapping.

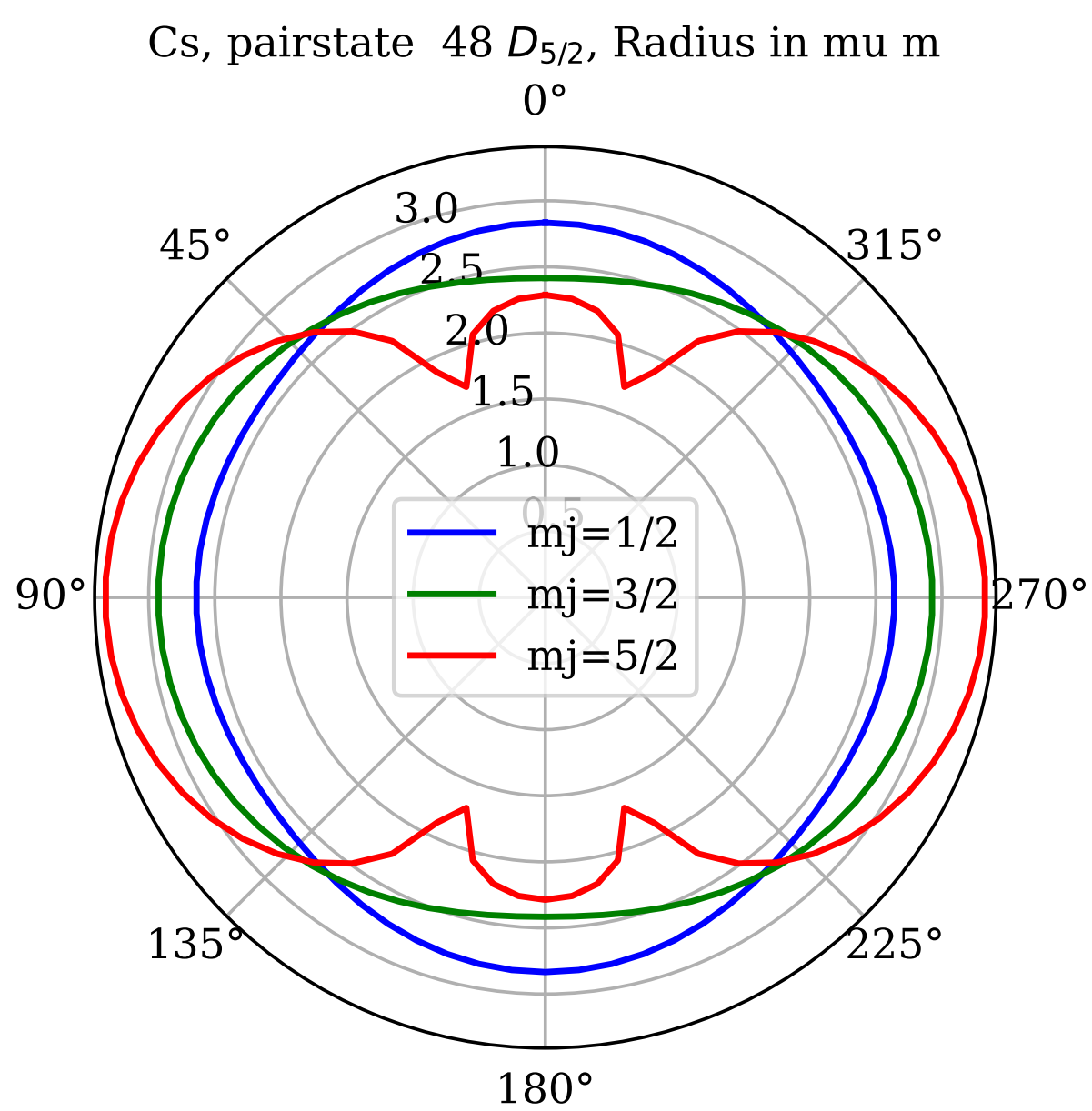

Figure 2: Blockade radii for $48D_{5/2}$ at different angles from 0° to 360°. The blue, green, and red curvescorrespond to total angular momentum magnetic quantum numbers 1/2, 3/2, and 5/2, respectively.

Response to Reviewer C

Comment C0 — *Authors have addressed the queries.*

References

[1] Nikola Šibalić, Jonathan D Pritchard, Charles S Adams, and Kevin J Weatherill. Arc: An open-source library for calculating properties of alkali Rydberg atoms. *Comput Phys Commun*, 220:319–331, 2017.

Time series learning in a many body Rydberg system with emergent collective nonlinearity

Authors address a very interesting avenue of Rydberg systems exhibiting emergent phenomena for time series prediction.Lorenz and temperature data are used to demonstrate enhanced learning capability in a many body system. The problem being addressed is of interest to a broad audience. However, the manuscript still lacks clear and sound physical interpretations of the obtained results. Before recommending for publication, below are my queries,

- The introduction is well written but mininal emphasis is given to the nonlinearity and Rydberg systems exhibiting the emergent dynamics.
- Its better to have subnames to figures for more clarity. Otherwise, its unclear at times which figure is referred.
- Authors mention fig 2b is outside bistable regime and c and d are in bistable region. But doesn't explain or justify the spectral features.
- Authors state "no nonlinear activation is usedd in this readout layer, so all non linear processing of input orignates from the dynamics of the Rydberg vapor". This statement requires a clearer explanation.
- In the Methods section, a two-level model is used to describe the nonlinear dynamics. This is a very crude approximation for a thermal Rydberg EIT system. Ref. 33 clearly establishes that two-level dynamics cannot fully capture the emergent collective phenomena. The limitations of this approximation should be explicitly discussed.
- The authors do not include Doppler averaging or the role of different velocity classes involved in the interaction that leads to an effective many-body global coupling. The implications of neglecting velocity-dependent effects on the collective dynamics and learning performance should be discussed.
- In the Methods section on experimental analysis of relaxation times, the fitting function is written as $A\exp\{(-t/\tau) + B\}$. It is unclear whether the constant B is inside the exponential. If so, the interpretation of the extracted relaxation times needs clarification.
- Discussion section is relatitvely weak in justifying the results.
